# Understanding the Stability of Poorly Covered Pickering Emulsions Using on‐Chip Microfluidics

**DOI:** 10.1002/advs.202409903

**Published:** 2025-02-02

**Authors:** Xuefeng Shen, Chang Chen, Berend van der Meer, Thomas E. Kodger, Uddalok Sen, Siddharth Deshpande, Jasper van der Gucht

**Affiliations:** ^1^ Laboratory of Physical Chemistry and Soft Matter Wageningen University and Research Stippeneng 4 Wageningen 6708 WE The Netherlands

**Keywords:** pickering emulsions, microfluidics, droplet coalescence, stabilization, poorly‐covered

## Abstract

Particle‐stabilized emulsions, also known as Pickering emulsions, have shown promise in areas that require long‐term stability with minimum use of surfactants. While most work has focused on densely covered Pickering emulsions, such emulsions are known to retain stability even when the interfaces are sparsely covered with particles. Here, the formation, dynamics, and stability of poorly covered model Pickering emulsions are studied in a controlled manner by utilizing a microfluidic platform. The formed Pickering emulsions remain highly stable, over at least 12 h, even with a surface area coverage below 3%. By directly visualizing the droplet interface at various stages, the exceptional stability is attributed to the highly spatially heterogeneous distribution of adsorbed particles which exclusively form particle bridges at the contact point between the droplets. Remarkably, these bridges are assembled in the form of crowns between the droplet interfaces, as visualized by confocal microscopy. The assembly behavior of the adsorbed particles in response to hydrodynamic forces and the formation of non‐uniform particle distribution are discussed by analyzing the different forces present during emulsification, corroborated by numerical simulations. In conclusion, using a lab‐on‐a‐chip approach, this work provides further understanding toward the fabrication of Pickering emulsions via preferential interfacial localization of particles.

## Graphical abstract

This study introduces a microfluidic approach to investigate the formation, dynamics, and stability of Pickering emulsions. The results show that the adsorbed particles assemble into crown‐like structures between droplets, preventing coalescence at low particle coverage. The proposed method also paves the way for fabricating novel materials, using Pickering emulsions as templates through selective particle localization, driven by hydrodynamic forces.

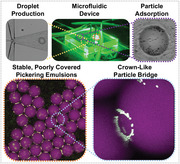

## 1 Introduction

The adsorption and assembly of colloidal particles at the droplet interface is a promising bottom‐up route for the fabrication of a broad class of nano‐ or micro‐structured materials.^[^ ^1^, ^2^, ^3^, ^4^, ^5^ ^]^ Since the early 20^th^ century, experiments have established that solid particles can stabilize droplets very effectively against coalescence, leading to the so‐called Pickering emulsions.^[^ ^6^, ^7^ ^]^ Because of the high desorption energy, the adsorption of particles at the liquid‐liquid interface can be considered virtually irreversible.^[^ ^8^ ^]^ The formation of a dense layer of closely packed particles then leads to a steric barrier, which prevents the droplets from coalescing.^[^ ^9^ ^]^ Surprisingly, stable emulsions can be formed even when the droplet interface is only partially covered by particles, even at a coverage as low as 5%.^[^ ^10^, ^11^ ^]^ While this phenomenon may have important consequences for the preparation and application of Pickering emulsions, the origin of the enhanced stability of scarcely covered emulsion droplets is still poorly understood. Previous work has suggested that stabilization may be due to a bridging mechanism, where particles accumulate preferentially at the contact region between droplets,^[^ ^12^, ^13^, ^14^ ^]^ forming a patch of particles that interconnect the two droplets.^[^ ^15^, ^16^, ^17^, ^18^, ^19^ ^]^ This bridging patch may consist of a single monolayer of particles that is adsorbed on the interfaces of both droplets simultaneously, or a bilayer where each droplet has a layer of particles that interlock to form a stabilizing structure. Another possible stabilization mechanism is the formation of a network structure at the contact region.^[^ ^20^ ^]^ However, the precise structure of the bridges between the droplets, the mechanism of their formation, and their role in stabilization, remain unclear. Moreover, it is not understood how the bridges form and why such a small amount of adsorbed particles accumulate preferentially in the contact zone between droplets. Previous work has focused on the examination of emulsions in a stationary state after their preparation, which disregards the dynamic behavior of the particles during emulsification and does not provide insight into mechanisms underlying the bridge formation. To obtain a better understanding, it is necessary to follow the emulsification process, including the dynamics of the particles, in situ.

Conventional emulsification methods, such as rotor‐stator homogenization, high‐pressure homogenization, and sonication, are widely used in the preparation of Pickering emulsions.^[^ ^21^ ^]^ Nonetheless, these methods produce droplets in an uncontrolled manner, hindering a systematic analysis of specific properties in a uniform population.^[^ ^22^ ^]^ Microfluidic emulsification enables precise droplet size control, uniform particle coverage, preservation of the integrity of delicate particles, and in situ observation, thus facilitating quantitative analysis in minute sample volumes.^[^ ^23^, ^24^ ^]^ However, creating Pickering emulsions in microfluidic channels is challenging because of channel clogging caused by particle aggregation, constraints related to the wettability of materials employed in microfluidic device fabrication, and the low hydrodynamic forces in microchannels, which are often insufficient for particles to surpass the adsorption energy barrier. So far, research on Pickering emulsions using microfluidics is primarily focused on harnessing the benefits of microfluidics in terms of controlling droplet size and homogeneity.

In this work, we use microfluidics to efficiently generate particle‐stabilized emulsions in a controlled manner. Importantly, our on‐chip setup allows us to visualize the underlying dynamics during production as well as after collection, to study particle accumulation and bridging, associated particle/fluid flows, and their effect on droplet coalescence. With this approach, we reveal that Pickering emulsions retain long‐term stability with estimated coverage less than 3%, owing to the preferential accumulation of particles at the droplet interface. By collecting these particle‐laden droplets in a collection chamber and using fluorescence microscopy, we show that particle bridges are predominantly formed at the droplet contact regions under low‐particle coverage conditions, exhibiting a rich variety of architectures, including monolayers, bilayers, or a network‐like multilayer. Remarkably, 3D reconstruction from confocal fluorescence imaging reveals a crown‐like structure of the bridging particles, that ultimately provides high stability to these Pickering emulsions. Moreover, experimental results highlight the significant impact of external fluid flow on the spatial accumulation of particles at the interface, as validated by numerical simulations. Our results thus provide strong‐evidence that the stabilization of Pickering emulsions with low‐particle coverage is due to particle bridges and further reveals their architecture and formation process.

## 2 Results and Discussion

### 2.1 Controlled on‐Chip Production of Pickering Emulsions

**Figure 1:**
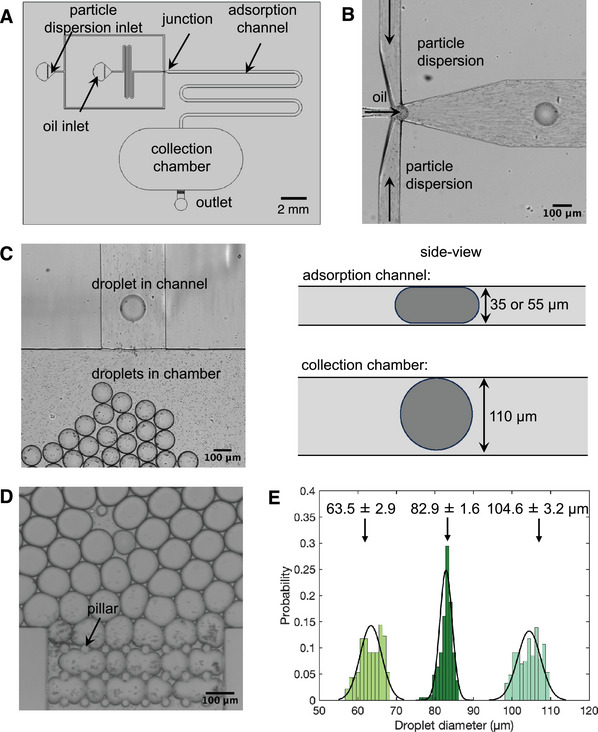
Monodisperse Pickering emulsion production and collection using on‐chip microfluidics. A) Schematic of the microfluidic device consisting of inlets for particle dispersion and oil phase, a flow‐focusing junction, a post‐junction particle adsorption channel, a collection chamber, and an outlet. B) Dodecane‐in‐water droplets are generated at the flow‐focusing junction, where the continuous phase contains 0.125 wt% polystyrene particles. C) The multi‐height microfluidic device ensures that the droplets transform from a pancake shape to a spherical shape after entering the collection chamber. Side‐view schematic comparing the variation of the droplet shape in the channel and in the chamber. D) The pillars at the outlet are designed to allow only the particles to flow out, while confining the droplets within the collection chamber. E) Normalized frequency histograms of different batches of collected droplets denoting uniform size distribution in each case; *N* ⩾ 186 for each histogram. Solid lines are Gaussian fits to the individual histograms.

We started with designing a lab‐on‐a‐chip system to ensure efficient production, collection, and visualization of Pickering emulsions. The schematic of our microfluidic device for generation and collection is shown in **Figure** **1**A. A conventional soft‐lithography method was used to manufacture a multi‐height polydimethylsiloxane (PDMS)‐based microfluidic device (see Experimental Section for fabrication details). We used 1 µm radius polystyrene particles as Pickering agents to create oil (dodecane)‐in‐water Pickering emulsions with droplet sizes ranging from 60–110 µm through a flow‐focusing approach. The polystyrene particles were functionalized with carboxylate groups to enhance stability and dispersibility in the aqueous solution. A typical experiment involves producing a continuous stream of dodecane droplets, which then flow through the post‐junction adsorption channel with the continuous aqueous phase containing the polystyrene particles, as depicted in Figure 1B and Video S1 (Supporting Information). While the flow velocities of the continuous phase and dispersed phase streams primarily governed the droplet size, the channel dimensions also dictated the minimum droplet volume, setting the lower limit on the droplet size. For generating droplets with diameters ranging from 80 to 110 µm, we used devices with a channel height of 55 µm. To obtain smaller droplets (less than 70 µm), we used devices with a channel height of 35 µm. To ensure that the droplets entering the collection chamber were not physically squeezed and remained spherical, the height of the collection chamber was designed to be larger and was kept at 110 µm for all devices, (Figure 1C). The pillars in the outlet allowed excess particles to flow out while retaining particle‐laden droplets within the collection chamber, as shown in Figure 1D. Control of droplet size and monodispersity is important for quantitative characterization of the stability of Pickering emulsions. Figure 1E shows histograms of three batches of droplets formed in the size range of 60–110 µm. The coefficient of variation ranged between 2% and 5% of the mean, reflecting a monodisperse size distribution in each case. This protocol thus allows us to produce monodisperse, spherical oil‐in‐water droplets in a controllable manner, with their interface coated with Pickering particles.

### 2.2 On‐Chip Particle Adsorption and Droplet Collection

Although the adsorption of particles at the oil‐water interface is thermodynamically favored, this process is neither spontaneous nor rapid for colloidal particles.^[^ ^25^ ^]^ This is mainly because most particles carry surface charge to prevent colloidal aggregation, and charged particles approaching a fluid‐fluid interface can experience an image‐charge force, which repels the particles from breaching the interface.^[^ ^26^ ^]^ Additionally, there also exists an electrostatic repulsion force between the negatively‐charged particles and the negatively‐charged hydrophobic/water interface.^[^ ^27^ ^]^ Thus, sufficient hydrodynamic forcing is necessary for the particle to breach the droplet interface.^[^ ^28^, ^29^, ^30^ ^]^ The zeta potential of the polystyrene particles in water was measured to be –28.4 ± 2.0 mV (Figure S1, Supporting Information), indicating the existence of repulsive forces between the particles and the droplet interfaces. Therefore, forces that overcome this repulsion are necessary for the adsorption of particles to the interfaces.

**Figure 2:**
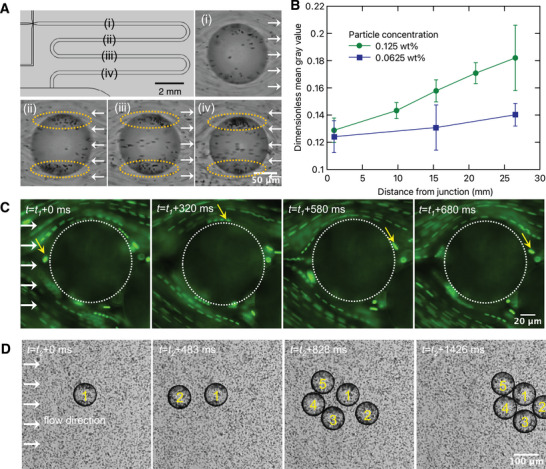
Visualization of particle adsorption and droplet bridging processes. A) Typical particle adsorption process as the droplet flows down the adsorption channel. Particles can be seen as black dots. The arrows represent the direction of the external fluid flow, while the dashed ellipses show the asymmetric accumulation of particles at the droplet surface. Particle concentration is 0.125 wt%. B) Dimensionless droplet mean gray value as a function of the distance from the production channel for two different particle concentrations. The increasing dimensionless mean gray value denotes an increasing amount of adsorbing particles. Data are presented as mean ± standard deviation; *N* = 5 droplets for each data point. C) Fluorescence microscopy images showing the trajectories of free particles moving past the droplets in the collection chamber. The white dotted circles and the yellow arrows indicate the droplet outline and the location of a representative particle over time, respectively. Particle concentration is 0.125 wt%. *t*_1_ refers to the start of the video recording. D) A time‐lapse sequence showing the formation of a typical droplet cluster in the collection chamber; *t*_2_ refers to the start of the video recording. White arrows in (C) and (D) indicate the direction of the outer flow. Particle concentration is 0.125 wt%.

**Figure** **2**A shows a time‐lapse of particle adsorption as the droplets flow downstream of the channel. Right after droplet formation, only a few particles could be seen adsorbed on the droplet surface. However, as the droplet flowed downstream, there was a continuous and notable increase in the number of adsorbed particles. These results suggest that, despite the laminar flow regime, relatively low fluid velocities in the range of millimeters per second, and a moderate repulsive force, the particles were able to breach the oil‐water interface and form Pickering emulsions. It is also worth noting that by the time the droplets exit the adsorption channel, their surface is still only partially covered by particles. While the observed continuous particle adsorption at the interface is somewhat expected, we made a surprising observation in terms of the spatial distribution of the adsorbed particles: the majority of the adsorbed particles accumulated at the droplet equator in a non‐uniform fashion, forming a bipolar distribution orthogonal to the flow direction, as indicated by the dashed ellipses in Figure 2A. This contrasts with surfactant‐stabilized droplets, where stabilizers typically accumulate at the rear of the droplets.^[^ ^31^ ^]^ The possible cause and the impact of uneven particle distribution on the formation of particle bridges between droplets and their stability will be discussed in subsequent sections. Figure 2B illustrates the time‐dependent particle adsorption at the droplet interface in terms of an increase in the dimensionless mean gray value (see Experimental Section for details), an indicator of the number of adsorbed particles. The distance from the production junction is directly proportional to the time available for adsorption because the flow rate is kept constant during these measurements. Thus, plotting the dimensionless mean gray value as a function of the distance from the production junction directly demonstrates the particle adsorption process, observed for two different particle concentrations, as the droplets move downstream.

After the droplets enter the wider and higher collection chamber, the increase in the cross‐sectional area leads to a pronounced decrease in the flow velocity. As a result, the hydrodynamic forces reduce as well, which should prevent the particles from penetrating the droplet interface. To verify this, the flow of the particles around the collected droplets was recorded. We captured particle traces flowing around the droplets, where the particles could be seen circumventing the droplets without any interfacial adsorption (Figure 2C; Video S2, Supporting Information). This observation remained consistent even after increasing the flow velocity by doubling the continuous phase inlet pressure. Such an observation likely reflects the significantly lower hydrodynamic forces in the collection chamber as compared to those present in the adsorption channel, thus preventing particles from overcoming the adsorption energy barrier. In the collection chamber, these droplets collide during flow, forming clusters that remain connected to each other. Figure 2D illustrates the formation process of a droplet cluster in the collection chamber, with new droplets arriving from the left side and colliding with the existing droplets. For example, droplet 2 physically contacts droplet 1 due to the fluid flow, and stays connected without coalescence. Three more droplets follow, eventually creating a cluster of five droplets within a second. Thus, individual poorly covered Pickering emulsion droplets are observed to efficiently form stable, non‐coalescing clusters in an on‐chip setting. We then focused on characterizing the thus‐formed Pickering emulsion droplets.

### 2.3 Microscopic Characterization of the Collected Pickering Emulsions

**Figure 3:**
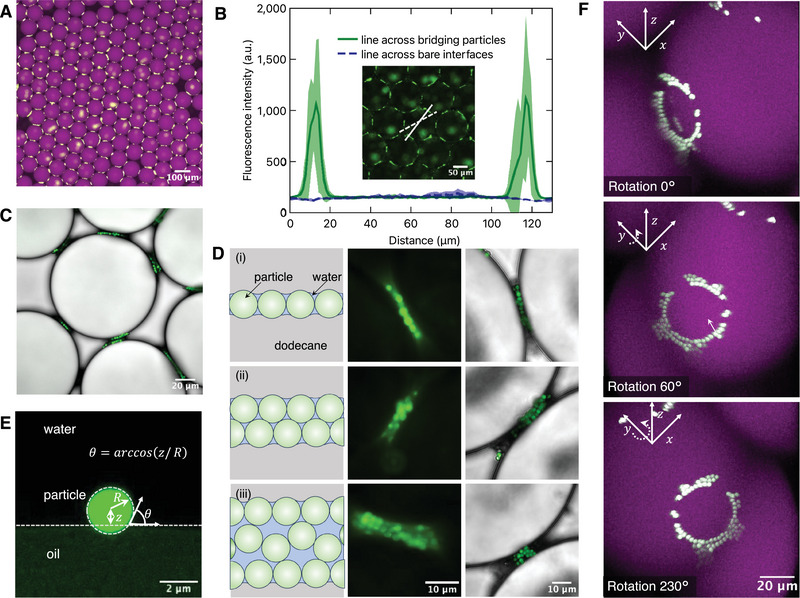
Particle bridges with crown‐like structure stabilize poorly covered Pickering emulsions. A) Two‐channel fluorescence micrograph of collected Pickering emulsions stabilized via particle bridges. The fluorescently labeled polystyrene particles appear light green, while the dodecane droplets doped with 250 µM Nile red are shown in magenta. B) Fluorescence intensity profiles across the interdroplet contact region and the bare interfaces, corresponding to the lines drawn in the inset figure. The interdroplet contacts show two sharp peaks corresponding to the particle bridges, and correspond well with the dip in the droplet fluorescence, indicating the presence of particles at the interface; the bare interfaces show a flat profile indicating an absence of particles. Data are presented as mean (solid lines) ± standard deviation (shaded areas); *N* = 5. C) Confocal micrograph of collected Pickering emulsions showing the exclusive presence of particles at the droplet contact points and also the variety of particle bridges that can be formed. D) The poorly covered Pickering emulsions can be stabilized by three types of particle bridges: i) a monolayer, ii) a bilayer, and iii) a multilayer consisting of aggregated particles. The three columns respectively depict schematic views (not to scale), epifluorescence micrographs, and confocal fluorescence micrographs. E) Confocal fluorescence micrograph showing a particle of 1 µm radius straddling the droplet interface and the extraction of necessary parameters for calculating the contact angle. F) 3D reconstruction of the fluorescence confocal images reveals the nature of the particle bridges in further detail. Strikingly, the particles are self‐assembled in the form of a crown at the interdroplet contacts. Particle concentration is 0.125 wt%.

Having established a microfluidic method to produce and collect Pickering emulsions, we set out to study the characteristics of the Pickering emulsions collected downstream. **Figure** **3**A shows epifluorescence micrographs of one such batch of collected Pickering emulsion droplets. As can be observed, the particles showed a highly non‐homogeneous distribution revealing two distinct regions: particle‐rich regions corresponding to contact points between Pickering emulsion droplets and particle‐free bare interfaces. These two regions can be further distinguished by plotting the fluorescence intensity profiles along a line across the bridging particles and the bare interfaces, as shown in Figure 3B. The intensity profile for the line crossing the bare interfaces exhibits minimal variation and a very low intensity, indicating the absence of any adsorbed particles. On the other hand, the intensity profile for the line through the interdroplet contacts shows two clear high‐intensity peaks at the droplet interface corresponding to the high density of adsorbed particles at the interdroplet contact space.

To gain physical insight into the stabilization mechanisms, we conducted further microscopy studies and took a closer look at the particle bridges. We used confocal fluorescence imaging to reveal the nature of the particle bridges. Figure 3C clearly illustrates the preferential spatial localization of particles at the contact region. The particles tend to accumulate in the contact region close to another droplet, leaving the remaining part of the droplet interface completely exposed. We observed a rich variety of particle bridges that could be primarily categorized into three types (Figure 3D): i) monolayers – a single particle layer shared by both the bridging interfaces; ii) bilayers – regions formed with a monolayer of adsorbed particles at each interface that interlock; iii) multilayers – bridges with multiple particle layers forming a network‐like structure. While monolayers and bilayers were more prominent, multilayers were relatively rare, and we consider them to be episodic under low particle coverage. Multilayer particle bridges are likely formed when two droplets trap free particles while approaching each other. Regardless of their type, these particle bridges effectively isolated the interfaces of adjacent droplets, thereby preventing coalescence. We also conducted experiments to determine whether the pH of the continuous phase affected the formation of particle bridges. We observed efficient particle bridges independent of the pH (Figure S2, Supporting Information), indicating that particle bridge formation is not primarily governed by electrostatic forces.

The micron‐sized particles we employed allowed us to directly measure the apparent contact angle of the adsorbed particles at the droplet interface, as shown in Figure 3E. Given the radius of the particle, we calculated the apparent contact angle of the particle at the interface by measuring the distance from the center of the particle to the interface, employing the equation: θ=arccos(z/R),^[^ ^32^ ^]^ where *R* is the radius of the particle and *z* is the distance between the center of the particle and the interface. The contact angle of the particles in equilibrium state for our system was found to be 79° ± 2° (*N* = 5). In Pickering emulsions stabilized by a particle monolayer, maintaining an appropriate particle contact angle is crucial for stability. Recent research indicates that when the contact angle of a particle exceeds 90°, adsorbed particles are prone to promoting coalescence, as a result of the overlap of the two interfaces in such a circumstance.^[^ ^33^ ^]^ The measured contact angle is thus in good agreement with this reasoning, given the stability of the formed emulsions.

For a more in‐depth understanding of the nature of the particle bridges, we performed a 3D reconstruction of the collected droplets using multiphoton confocal fluorescence microscopy. The 3D perspective in Figure 3F distinctly illustrates the assembly of adsorbed particles into a crown structure (Video S3, Supporting Information). This crown structure is not necessarily uniform, comprising not only of a single ring of particles but also multilayered particle arrangements or with regions without particles. The reconstruction also reveals that the bridging particles, when observed from viewpoints at different rotation angles around the *y*‐axis, may appear as different types of bridges, as shown in Figure 3F. With a measured contact angle θ = 79°, particle radius *r*_*p*_ = 1 µm, crown radius *r*_*c*_ = 15 µm, and droplet radius *r*_*d*_ = 50 µm, and taking a simple case of the crown structure consisting of a single ring of particles, we estimated the particle coverage of monolayer‐packed droplets to be 2.72% (Note S1, Supporting Information). Our findings indicate that in the case of droplets packed in a monolayer configuration, crown‐shaped structural bridging particles exhibit significantly lower coverage (2.72%) compared to disk‐shaped structural bridging particles (11.80%) of identical crown radius (Note S1, Supporting Information). Thus, the 3D structure of the bridging particles, in the form of hollow crown, plays a crucial role in the long‐term stability of Pickering emulsions with poor particle coverage. We would also like to note that, according to Gibbs adsorption law, the surface pressure can be estimated as Π_*s*_ ≈ Γ*k*_*B*_ *T*, where Γ is the number of particles per unit area. Based on Note S1 (Supporting Information), droplets with a diameter of ≈100 µm have ≈300 particles adsorbed at their surfaces; thus the resulting reduction in the interfacial tension is in the order of 10^−11^ N m^−1^, well below the detection limit of tensiometric methods.^[^ ^30^ ^]^ Therefore, the reduction of interfacial tension caused by the adsorbed particles is not considered in this work.

### 2.4 Coalescence Stability of Poorly Covered Pickering Emulsions

After revealing the microstructure of the particle bridges, we next sought to investigate their effect on the emulsion stability. While numerous factors impact the stability of emulsions, the physical destabilization of emulsions can proceed through two distinct mechanisms: Ostwald ripening and coalescence.^[^ ^34^ ^]^ Ostwald ripening arises from the diffusion of the dispersed phase through the continuous phase, and leads to an increase in the average droplet size, with a concomitant decrease in their number. Owing to the extremely low solubility of dodecane in water, Ostwald ripening of dodecane droplets in our experiments should be very slow, on the order of weeks.^[^ ^35^ ^]^ Additionally, because the produced droplets are already very monodisperse, the Laplace pressure difference responsible for Ostwald ripening is minimal. Thus, in this study, we can safely ignore the influence of Ostwald ripening on emulsion destabilization and focus only on coalescence.

**Figure 4:**
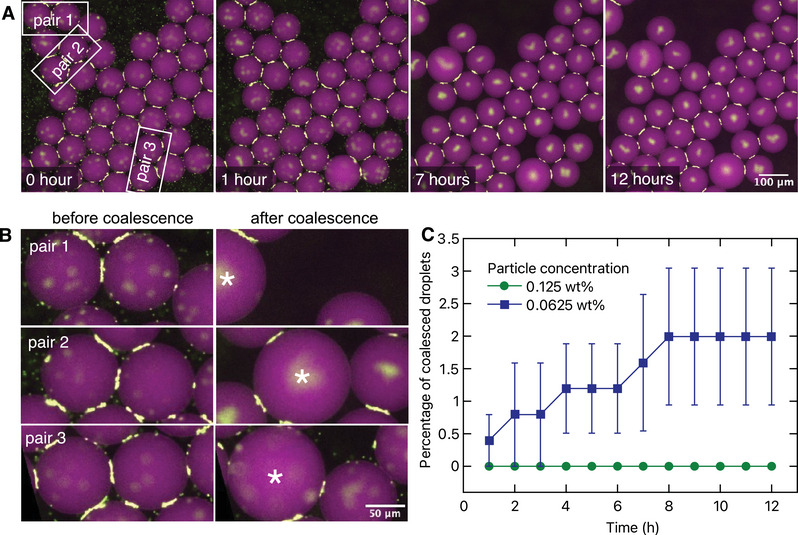
Poorly covered Pickering emulsions remain highly stable against coalescence. A) Typical droplet coalescence events of collected Pickering emulsions. White rectangles indicate the droplet pairs undergoing coalescence. Particle concentration is 0.0625 wt%. B) Zoomed‐in views showing droplet pairs before and after coalescence; coalesced droplets are marked with an asterisk. C) Percentage of coalesced droplets as a function of time for two different particle concentrations. Data are presented as mean ± standard error; *N* = 3 independent experiments in each case.

To determine the minimum particle concentration required for long‐term stability, we generated Pickering emulsions with particle concentrations of 0.0625 and 0.125 wt%, and monitored coalescence events over the course of 12 h. **Figure** **4**A shows a typical time‐lapse sequence within a selected area of the collection chamber, revealing just three coalescence events over the entire duration. Due to the long time interval (1 min) between the frames, discerning whether the coalescence process of particle‐covered droplets differed significantly from that of bare droplets was not possible in our experiments. Close‐up views of droplet pairs before and after coalescence are presented in Figure 4B. We quantified the percentage of coalescence events over 12 h (0% reflecting no coalescence, 100% reflecting all the droplets coalesced, see Experimental Section for details), which is shown in Figure 4C. As can be seen, for a low enough particle concentration (0.0625 wt%), sporadic coalescence events can be observed before the system reaches an equilibrium stage, with the average percentage of coalesced droplets to a mere 2% (Video S4, Supporting Information). On the other hand, upon doubling the particle concentration (0.125 wt%), coalescence is completely suppressed right from the beginning for at least 12 h. It is worth noting that even a particle concentration of 0.125 wt% in the dispersed phase is exceptionally low and an overestimation because, contrary to traditional emulsification techniques where all particles eventually adsorb to the interface, the majority of free particles in the continuous phase exit the device in microfluidic emulsification, resulting in a significantly‐reduced effective particle concentration. In conclusion, the preferential particle accumulation at the interdroplet contact physically prevents coalescence events, giving poorly covered Pickering emulsions long‐term stability.

### 2.5 Understanding the Formation of Particle Bridges Between Droplets

Our aforementioned experimental observations indicate that, in Pickering emulsions with low particle coverage, preferential localization of the particles at the interdroplet contact regions plays a crucial role in suppressing coalescence and maintaining long‐term stability. These bridging particles are subject to various forces, such as capillary forces, electrostatic forces, and hydrodynamic forces, which can facilitate as well as hinder bridge formation. Therefore, we further investigated the reasons behind the tendency of these adsorbed particles to accumulate in regions adjoining droplet interfaces, forming particle bridges that prevent the coalescence of droplets.

We first focused on the interaction forces between the particles, considering two scenarios: particles adsorbed at the same interface and particles shared between two neighboring interfaces. As the particles used in this study carry a charge, there exists an electrostatic repulsion force and a capillary attraction force between two particles adsorbed on the same interface.^[^ ^36^, ^37^ ^]^ We observed that adsorbed particles that are not part of a bridge between two droplets attract each other and form clusters (Figure S3, Supporting Information), indicating that the capillary attraction force dominates over the electrostatic repulsion force. For particles adsorbed at both interfaces of a liquid film with varying spatial thicknesses, an additional form of capillary force arises from the difference between the local contact angle and the equilibrium contact angle.^[^ ^38^ ^]^ Particles with contact angles closer to 90° (present scenario) tend to accumulate in the thinnest regions of the film between the two interfaces and prevent the contact of two interfaces. For example, when a dimpled liquid film forms between two droplets, this capillary force drives the particles to accumulate at the edge of the dimple, because the liquid film is thinnest there.

**Figure 5:**
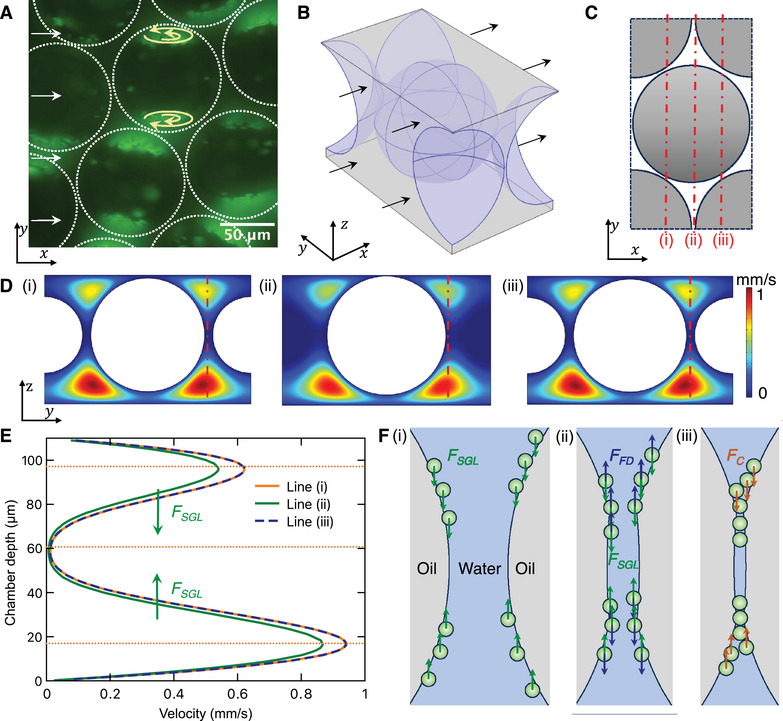
External fluid flow largely determines the observed particle bridges between the droplets. A) Bottom view of the collected Pickering emulsions showing that the adsorbed particles tend to accumulate at the poles orthogonal with respect to the external fluid flow (direction indicated by white arrows) to form a bipolar structure. The white circles indicate the droplet interfaces, while the yellow arrows indicate the circulation direction of the accumulated particles at the poles. Particle concentration is 0.25 wt%. B) Geometry of the computational domain, arrows indicate the direction of external fluid flow. C) Schematic top‐view, showing the location of the cut planes (red lines). D) Velocity distribution across the cut planes of the computational domain, showing that the high‐velocity regions occur at the narrowest sections adjacent to the droplet. The cut lines used in further calculations are shown in red. E) Velocity profiles along the cut lines in (D). From top to bottom, particles dispersed between the first and the second, and the second and the third dashed orange lines are subject to downward and upward lift forces, respectively. F) Schematic view of proposed crown structure formation: i) The velocity gradient lift force (*F*_*SGL*_) propels adsorbed particles toward droplet contact region; ii) Interdroplet film drainage exerts a force (*F*_*FD*_) on the particle opposite to the lift force; iii) The bridging particles remain at the periphery of the water film, while the nearby particles are subjected to attractive capillary forces (*F*_*C*_), and together with the bridging particles, assemble into a crown structure.

The hydrodynamic forces during emulsification can also profoundly impact the accumulation of particles in the contact region. Previous research has emphasized the necessity of a substantial hydrodynamic force that drives the droplets closer to each other to form bridged structures; emulsions subjected to mild agitation, such as by manual shaking, only showed individual and inadequately covered droplets.^[^ ^16^ ^]^ Nevertheless, there is currently a lack of understanding of how the effects of the external flow influence the accumulation of particles and the formation of particle bridges. The microfluidic technique used in this study provided us with a platform for the experimental investigation of the particle accumulation. We designed experiments to verify how adsorbed particles respond to an external flow field. A continuous phase with a higher particle concentration (0.25 wt%) was used to enhance the visualization of adsorbed particles. Since dodecane is lighter than water, droplets float in the collection chamber when their diameter is less than the chamber height. Note that these floating droplets were still separated from the upper chamber wall by the particles adsorbed at the droplet interface. Subsequently, we flushed the prepared droplets with MilliQ water, varied the flow rate, and recorded the behavior of the adsorbed particles. **Figure** **5**A (also see Video S5, Supporting Information) illustrates the resulting accumulation of the adsorbed particles at the droplet interfaces perpendicular to the main flow direction, confirming the influence of the external flow field. These accumulated particles further showed a circular motion at the poles. As mentioned earlier, such bipolar accumulation in the direction orthogonal to that of the fluid flow was also observed for the droplets flowing in the adsorption channel before forming clusters in the collection chamber, as shown in Figure 2A. Having observed these similar phenomena, we further explored the underlying causes that could contribute to their occurrence.

To visualize the flow field around the droplet, we performed a numerical simulation using the finite element method. For simplification, we omitted the influence of droplets and particles on the entire flow field, treating the droplets as rigid‐solid spheres in the fluid and focusing solely on obtaining the flow field around the droplets. For the boundary conditions of the droplet (sphere) surface, we compared results using both slip and no‐slip boundary conditions. Details of the computational domain and boundary conditions are discussed in Note S2 (Supporting Information). The results indicated that velocities were slightly higher with the slip boundary, yet the trends in velocity distribution and changes remained consistent, with additional details in Figure S4 (Supporting Information). It is recognized that even minute amounts of impurities can result in an immobile liquid/liquid interface.^[^ ^39^ ^]^ Consequently, in our study, since adsorbed particles immobilize the droplet interface, the droplets in the collection chamber behave as if possessing a no‐slip interface. Therefore, we apply a no‐slip boundary condition in our simulations, setting the velocity at the droplet surfaces to zero. We solved the Navier–Stokes equations for single‐phase flow to simulate the experimental scenario. Figure 5B illustrates a single representative bi‐periodic computational domain of hexagonally packed droplets. A pressure difference was set between the inlet and outlet boundaries, generating a stationary flow in the direction indicated by the arrows. Although the actual pressure difference was not measured, the calculated flow speed, which is on the order of millimeters per second, corresponds closely to our experimentally‐measured particle flow speed. Figure 5C shows the top‐view of the computational domain and the location of three cut‐planes. Figure 5D displays the velocity field across three cut planes, suggesting sharp velocity gradients. Figure 5E shows the velocity profiles along the selected cut lines from Figure 5D. We selected the cut lines close to the droplet poles because of the proximity of the flow field to the particles adsorbed at the interface. In microfluidic channels, a particle experiences a shear gradient lift force, *F*_*SGL*_, which is attributed to the curved velocity profile. This shear gradient lift force is typically directed from the high‐velocity region toward the low‐velocity region,^[^ ^40^ ^]^ which is indicated by the arrows in Figure 5E. Thus, our proposed mechanism leading to the crown‐structured particle bridge formation is as follows: As droplets approach each other, high‐velocity regions form at the narrowest sections adjacent to the droplets, indicating flow acceleration due to the reduced cross‐sectional area. This results in a near‐parabolic velocity profile that generates a shear gradient force acting on the particles, directing them toward the poles of the droplet, as shown in Figure 5F‐i. As the droplets move even closer, the drainage of the thin water film between two interfaces exerts a force in the direction opposite to the lift force, as illustrated in Figure 5F‐ii. Subsequently, the particles in the thinnest region of the film contact the opposite film surface and become adsorbed to both, effectively bridging them. Then, as previously discussed, spherical particles adsorbed at both interfaces of a liquid film with spatially varying thickness experience an additional force in the form of capillary force. This force arises from the disparity between the local and equilibrium contact angles, pushing the particle toward a region where equilibrium is achieved at both interfaces. Ultimately, interface deformations caused by bridging particles generate a capillary attractive force, drawing neighboring adsorbed particles together to overlap the deformation and assembly into a crown, as shown in Figure 5F‐iii. Due to the interface confinement, the outer fluid flow, and the internal droplet flow, the accumulated particles circulate at the poles of the droplet. We visualized the droplet internal flow direction by introducing hydrophobic PMMA tracer particles inside the droplets. The observed results indicate the presence of internal circulation flow, as illustrated in Figure S5 and Video S6 (Supporting Information), and this circulation follows the same direction as observed for particles accumulated at the poles of the droplets shown in Figure 5A.

## 3 Conclusion

In this work, we have developed an on‐chip microfluidic platform for preparing and studying Pickering (particle‐stabilized) emulsions in a controlled manner. By subjecting the droplets to a prolonged particle‐rich laminar flow, we have demonstrated that monodispersed particle‐coated droplets can be produced and collected in a controlled manner. Our results demonstrate that Pickering emulsions can maintain long‐term stability (hours), even under conditions of very low particle coverage (<3%), due to the formation of particle bridges between droplets. Fluorescence confocal microscopy revealed mono‐, bi‐, or multi‐layered nature of the particle bridges. Furthermore, 3D reconstruction revealed that these particle bridges have a crown‐like appearance, thus stabilizing the droplet contact points with a very low amount of particles. The formation of the crown is related to a highly non‐uniform distribution of adsorbed particles at the droplet interface when droplets flow through the adsorption channel or are flushed by an external fluid in the collection chamber. Surprisingly, these particles accumulated at the poles of the droplets rather than at the rear, as is commonly observed for surfactant‐stabilized droplets. Using numerical simulations, we highlight the critical influence of hydrodynamic forces arising from the external fluid flow on the particle accumulation. Our findings thus highlight the significant influence of hydrodynamic forces on the assembly behavior of particles adsorbed at the droplet interface, an area that has received less attention compared to the effects of capillary and electrostatic forces. While the numerical simulations conducted here aim to provide a preliminary qualitative understanding of the phenomenon, a more extensive numerical model can be developed in future for quantitative analysis.

While our work addresses the stability of droplets under controlled laboratory conditions, further investigation can explore several aspects, such as the feasibility of industrial‐level production considering similar droplet generation principles already being used for large‐scale production,^[^ ^41^ ^]^ limitations arising due to low particle coverage, and whether sparse particle coverage can improve mechanical stability under shear forces^[^ ^12^ ^]^ or enhance the chemical stability of Pickering emulsions.^[^ ^3^ ^]^ In conclusion, the proposed method holds potential for fabricating novel materials using Pickering emulsions as templates through selective particle localization driven by hydrodynamic forces.

## 4 Experimental Section

### Materials

Aqueous solutions were prepared using MilliQ water with 18 MΩ resistivity (Milli‐Q, Merck Millipore). Dodecane (≥99%) was purchased from TCI Europe N.V. Polystyrene particles (L4530) and Nile Red were purchased from Sigma–Aldrich. Microfluidic accessories including tubing and PDMS coupler were purchased from Darwin Microfluidics.

### PMMA Particle Synthesis

Monodisperse, cross‐linked poly(methyl methacrylate) (PMMA) particles ( 0.6 µm in radius) were synthesized following methods presented in Refs. [42, 43, 44]. The particles were fluorescently labeled with Cyanine3 (Cy3), cross‐linked with ethylene glycol dimethacrylate, and sterically stabilized by the graft copolymer poly(12‐hydroxystearic acid). As the hydrophobic nature of these particles enabled their stable dispersion in dodecane, they were introduced only as tracers to visualize the internal flow within the droplet and not as Pickering particles, as they did not exhibit adsorption behavior at the interface.

### Fabrication of Microfluidic Devices

Microfluidic devices were fabricated using a mask‐less soft lithography technique. First, the photoresist (SU‐8 series, Kayaku) was spin‐coated onto a 76 mm diameter silicon wafer (Silicon Materials), where the spin speed and duration were adjusted to give a controllable uniform thickness. The microfluidic channel pattern was designed in AutoCAD 2023 (AutoDesk) and was exposed to the photoresist using the direct‐write photolithography system (MicroWriter ML 3, Durham Magneto Optics). A master was cured in the photoresist by UV light exposure. The uncured photoresist was removed using the developer propylene glycol monomethyl ether acetate (PGMEA, ≥99.5%, Sigma–Aldrich), leaving cured microstructures on the silicon wafer. To achieve channels and collection chambers of varying heights, the above process was repeated twice. SU‐8 50 was used for the first layer, which included inlet and adsorption channels, both set at heights of 35 and 55 µm, achieved through spin speeds of 3500 rpm and 2200 rpm respectively. SU‐8 100 was used for the second layer to form the collection chamber at a height of 110 µm with a spin speed of 2600 rpm. PDMS (Sylgard 184, Dow Corning) mixture of 10: 1 volumetric ratio of base to cross‐linker was poured over the wafer, and the air bubbles trapped during the mixing were removed by desiccating in a vacuum desiccator for 40 min, before baking for 3 h at 70 °C in an oven. The resulting solid PDMS layer, featuring the designed channels, was then peeled off from the wafer, and round holes with 0.5 mm diameter were punched at inlets and outlets through an rapid‐core microfluidic punch (Darwin Microfluidics). The PDMS channel layer, as well as a PDMS‐coated cover glass, were bonded together after treating with air plasma cleaner (PDC 32G, Harrick Plasma) for 60 s.

PDMS exhibits inherent hydrophobicity, which prevents the formation of either oil/water or water/oil/water emulsion. In order to enable such emulsion formation, the channels carrying the continuous (aqueous) phase in the microfluidic device should be rendered hydrophilic. Plasma treatment is a common method to hydrophilize PDMS surfaces.^[^ ^45^ ^]^ However, one drawback of this approach is that the effect of plasma treatment is temporary and thus not desirable in long‐term applications. Filling the channels with MilliQ water after plasma bonding has been found to provide long‐lasting hydrophilicity to the PDMS‐based microfluidic chips.^[^ ^46^, ^47^ ^]^ Here, hydrophilic PDMS devices that remain usable for weeks by employing plasma treatments and storing them in MilliQ water were produced. The results show that a plasma treatment at a power of 18 W for 60 s makes the surface sufficiently hydrophilic. Filling the treated PDMS devices with MilliQ water then allows them to maintain their hydrophilicity for at least 4 weeks, as shown in Figure S6 (Supporting Information).

### Microfluidic Experiments

MilliQ water, with dispersed 1 µm mean radius carboxylate‐modified polystyrene particles, as the continuous phase fluid was used. The continuous phase was diluted from the initial manufacturer‐supplied concentration of 2.5 wt% to the required concentration by adding MilliQ water with a pipette. Dodecane was the dispersed phase fluid, which was rendered fluorescent by the addition of the fluorescent dye Nile Red (250 µM). The flow rates of the fluids were independently controlled by a pressure controller (OB1 MK4, Elveflow) with a precision of 120 µbar.

### Imaging and Post‐Processing

Bright‐field and epifluorescence images were acquired using a inverted fluorescence microscope (Ti2‐Eclipse, Nikon) equipped with a illumination system (pE‐300ultra, CoolLED) and a high‐speed camera (Prime BSI Express sCMOS, Teledyne Photometrics). 2D confocal images were recorded on a confocal laser scanning microscope (C2, Nikon). 3D reconstruction based on confocal images was performed using a multiphoton confocal microscope (TCS SP8, Leica). Fiji (ImageJ) was used to process the acquired images. The fluorescently‐labeled polystyrene particles (Figures 2, 3, 4, and 5) were visualized using tetramethylrhodamine isothiocyanate (TRITC) filter sets, whereas the Nile Red‐labeled dodecane (Figures 3 and 4) and the Cyanine3‐labeled PMMA particles (Figure S5, Supporting Information) were visualized with the TLV‐TE2000‐GFP2 set. The TRITC filter set had an excitation wavelength of 543 nm and an emission wavelength of 593 nm, whereas the TLV‐TE2000‐GFP2 filter set featured an excitation wavelength of 482 nm and an emission wavelength of 520 nm. In Figure 2, the adsorption of particles was analyzed by measuring the gray value. The gray value of the particles is significantly different from that of the background and thus noticeable differences in gray values were observed when the number of adsorbed particles increased (Figure 2A). Figure 2B quantitatively shows the adsorption of particles by calculating the dimensionless mean gray value: *MGV*_dimensionless_ = (*MGV*_*background*_ − *MGV*_*droplet*_)/*MGV*_*background*_. In Figure 4, the coalescence of collected droplets was discussed. Epifluorescence microscopy images with ≈100 droplets were taken and the number of droplets was counted in each image. The percentage of coalesced droplets *N*_coal_ during the experiment was calculated by *N*_coal_ = (*N*_*t*_/*N*_0_) × 100, with *N*_*t*_ the number of coalesced droplets present at time *t* and *N*_0_ the initial number of droplets. *N*_coal_ equals 0 when no coalescence has occurred and increases with increasing coalescence events (Figure 4C).

### Zeta‐Potential Measurement

The net surface charge of the polystyrene particles was determined by measuring the zeta‐potential at 20 °C with a Zetasizer Nano (Malvern Instrument, Malvern, UK). To ensure clarity and to avoid multiple scattering, the aqueous phase containing the particles was diluted until transparent. The zeta‐potential was calculated from the average of five independent measurements.

### Contact Angle Measurement

Carboxylate‐functionalized polyethylene particles with a radius of 1 µm, which were fluorescently‐labeled with an excitation maxima of 480 nm and an emission maxima of 501 nm were used. Thus, these particles could be distinguished from the oil phase and the water phase using a confocal laser scanning microscope. This enabled us to capture the position of the particles at the interface and calculate the apparent contact angle (Figure 3E). To achieve refractive index matching, sucrose was added (Sigma–Aldrich) to the aqueous phase, which improved the resolution of the micrographs. A 50 wt% aqueous sucrose solution, which has a refractive index (≈1.42) similar to that of dodecane and a comparable surface tension (≈71 mN m^−1^) to that of water was prepared.

### Numerical Simulation of the Flow‐Field

Numerical simulation was performed to visualize and quantify the flow field around the collected droplets. The governing equations were solved by the finite element method using COMSOL Multiphysics 5.6, thus allowing for the estimation of the flow variables such as pressure and velocity. The problem was simplified to be a single‐phase stationary flow since the focus was on the flow field around the droplet. The governing equations for the steady 3D flow of a Newtonian fluid through an immobilized rigid‐body array are given by

(1) $$\rho \left(u\cdot \nabla \right)u=\nabla \cdot [-pI+\eta \left(\nabla u+{\left(\nabla u\right)}^{T}\right]\;$$

(2) ρ∇·u=0

where ρ, **u**, η, *p* and **I** are the density, velocity vector, dynamic viscosity, pressure, and identity tensor, respectively.

## Conflict of Interest

The authors declare no conflict of interest.

## Author Contributions

X.S., S.D., and J.v.d.G. conceived the research. X.S., C.C., and S.D. designed the microfluidic devices. X.S. and B.v.d.M. performed the 3D confocal microscopy experiments. B.v.d.M. synthesized the PMMA particles. X.S. performed the experiments and simulations. X.S., C.C., B.v.d.M., U.S., T.E.K., S.D., and J.v.d.G. discussed the results. X.S., S.D., and J.v.d.G. undertook the data interpretation and wrote the manuscript. All authors agree with the final version of the manuscript.

## Supporting information

Supporting Information

Supplemental Video 1

Supplemental Video 2

Supplemental Video 3

Supplemental Video 4

Supplemental Video 5

Supplemental Video 6

Supporting Data

## Data Availability

The data that support the findings of this study are available in the supplementary material of this article. Any additional data is available from the authors upon reasonable request.
