# Supplementary material for: Understanding the Stability of Poorly Covered Pickering Emulsions Using on‐Chip Microfluidics
Source: Adv Sci (Weinh). 2025 Feb 2;12(12):2409903. doi: 10.1002/advs.202409903 (PMC11948060; doi:10.1002/advs.202409903)
Supplement: Supplementary file 1 — Supporting Information [file ADVS-12-2409903-s008.pdf]

ADVANCED SCIENCE
Open Access

Supporting Information

for *Adv. Sci.*, DOI 10.1002/advs.202409903

Understanding the Stability of Poorly Covered Pickering Emulsions Using on-Chip Microfluidics

Xuefeng Shen, Chang Chen, Berend van der Meer, Thomas E. Kodger, Uddalok Sen, Siddharth Deshpande and Jasper van der Gucht**

Contents

1 Supplementary Notes **2**

2 Supplementary Figures **4**

3 Captions for Supplementary Videos **10**

1 Supplementary Notes

1.1 Supplementary Note 1: Estimation of particle coverage for monolayer-packed droplets

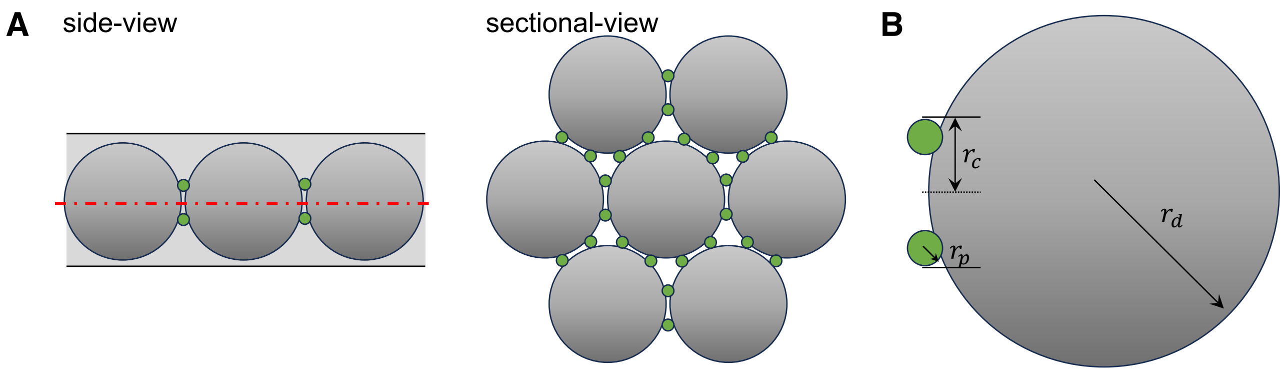

Schematic Figure S1: **Schematic diagram of monolayer-packed droplets. A** Side-view (left) of the collected droplets with a monolayer of adsorbed particles. A cross-sectional view (right) as seen when cut through the center of the droplet cluster (shown by red dotted line). **B** A diagram showing the three key radii: droplet, crown, and particle. The schematics are not to scale.

Considering the simplified case of a crown structure consisting of a single ring of particles, we estimated the particle coverage (ϕ) using measured contact angle (θ), crown radius (r_c), droplet radius (r_d), and particle radius (r_p) as

$$\phi = \frac{NS_c}{S_d} = \frac{N\pi \sin^2\theta\, r_c r_p}{4r_d^2}\,. \tag{1}$$

Here, N represents the number of crowns on each droplet, determined by the packing configurations of the collected droplets, S_d the surface area of the droplets, and S_c the cross-sectional area of the crown, and they can be calculated as

$$S_d = 4\pi r_d^2\,, \tag{2}$$

$$S_c = n\pi(\sin\theta r_p)^2 = \pi^2 \sin^2\theta r_c r_p\,. \tag{3}$$

Here, n is the number of particles per crown, which was estimated as

$$n = \frac{2\pi r_c}{2r_p} = \frac{\pi r_c}{r_p}\,. \tag{4}$$

For a monolayer of hexagonally-packed droplets discussed here, the value of n is 6, as shown in Schematic Figure S1A . With $\theta = 79°$, $r_p = 1$ μm, $r_c = 15$ μm, and $r_d = 50$ μm, we calculated the particle coverage to be 2.72%, corresponding to 283 particles adsorbed at the interface. Assuming the collected droplets form multilayered clusters, and not considering the boundary of the emulsion system for simplification, the value of n is 12 for both square and hexagonal dense-packing configurations. Consequently, the particle coverage is calculated to be 5.44%. In both scenarios, the estimated particle coverage remains remarkably low. If we consider these bridging particles to form a densely hexagonal packing disk, then the particle coverage area can be estimated by:

$$S_c = 0.9069 N\pi r_c^2 \sin^2(\theta) \tag{5}$$

The calculated particle coverage ϕ is 11.80%.

1.2 Supplementary Note 2: Computational domain

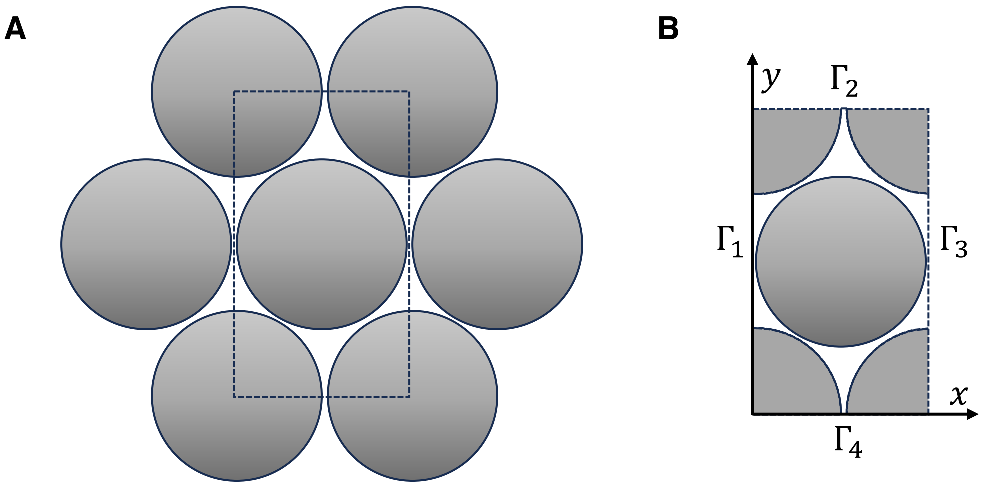

Schematic Figure S2: **A** Hexagonally-packed droplets. **B** A single representative bi-periodic domain.

The hydrodynamic interaction between the droplets, the channel walls, and the external fluid determines the flow field around droplets. We numerically-resolve this flow field in a bi-periodic domain, which allows for the flow problem with a large number of droplets to be transformed into a flow problem in a unit cell with a relatively small number of droplets. In this way, we can diminish finite size effects of the computational domain and solve the problem at a reasonable computational cost[1]. Schematic Figure S2 shows that the regular hexagonal droplet packing in this study can be represented by two droplets in a unit domain. With the flow being driven by the pressure drop between inlet Γ_1 and outlet Γ_3, the boundary conditions in this periodic domain can be expressed as

$$p_{src} = p_{dst} + \Delta p \,, \tag{6}$$

$$\mathbf{u}_{src} = \mathbf{u}_{dst} \,. \tag{7}$$

where the subscripts *src* and *dst* represent the boundaries Γ_1 and Γ_3, respectively. Another set of periodic boundary conditions was applied to the boundaries Γ_2 and Γ_4. The top and bottom boundaries are solid walls, which were described by the no-slip boundary condition.

It is well known that even trace amounts of impurities will immobilize the oil-water interface[2]. Consequently, we imposed a no-slip boundary condition on the surfaces of the droplets (spheres), assuming the velocity $\mathbf{u}$ at the droplet surfaces to be zero. From a modeling perspective, this assumption is considered a reasonable approximation, provided that the primary role of the spheres here is to emulate the impact of the droplet geometry on the overall flow field. The slip condition prescribes a no-penetration condition, $\mathbf{u} \cdot \mathbf{n} = 0$, where $\mathbf{n}$ is the unit normal vector. It implicitly assumes that there are no viscous effects on either side of the slip wall and hence, no boundary layer develops[3]. The velocity profiles obtained with the no-slip and slip boundary conditions are compared in Figure S4.

2 Supplementary Figures

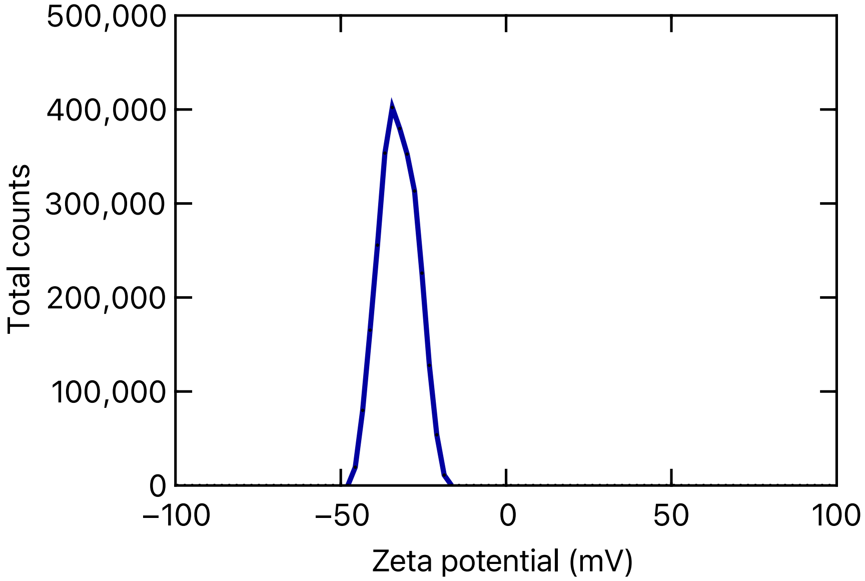

Figure S1: **Zeta-potential distribution profile for particles dispersed in Milli-Q water.** The aqueous phase containing the particles was diluted until a clear solution was obtained, in order to avoid multiple scattering. The average zeta potential of the particles was measured to be -28.4 ± 2.0 mV; $N = 5$ independent measurements.

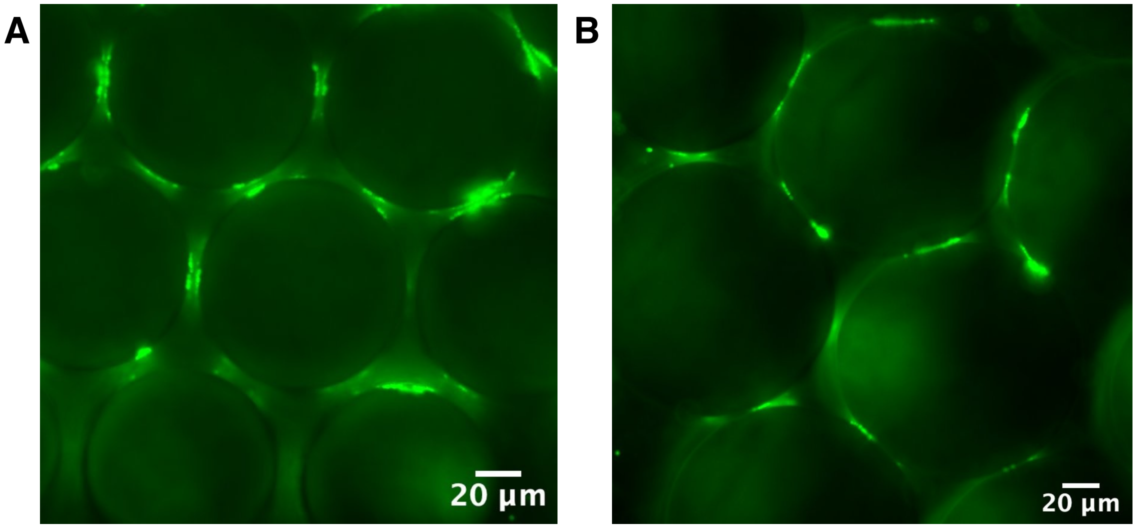

Figure S2: **Effect of pH variation on the formation of particle bridges between droplets.** Particle bridges are formed between droplets in a similar manner, regardless whether the pH of the continuous phase is 4 **(A)** or 10 **(B)**, indicating that the formation of particle bridges is largely independent of pH.

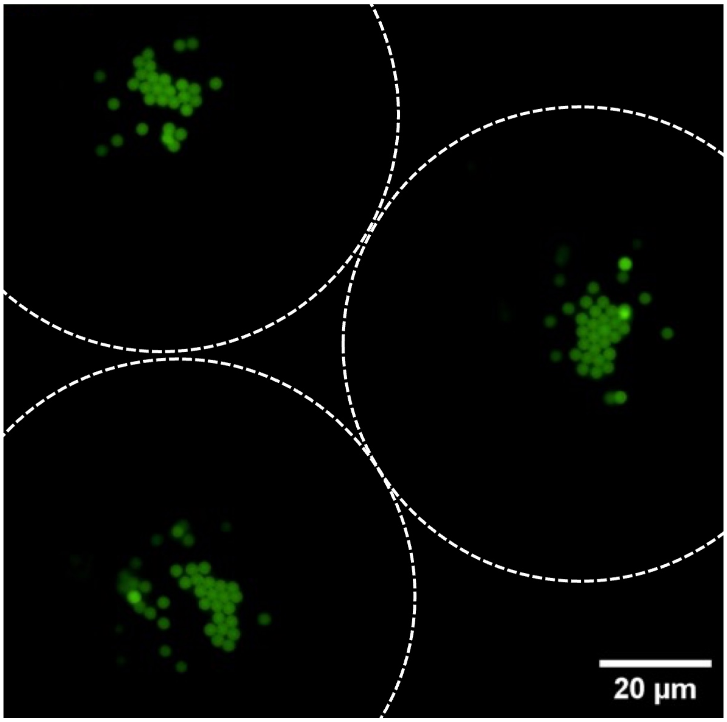

Figure S3: **Aggregation of adsorbed particles at the droplet interface.** After collecting the droplets (within a few hours), the non-bridged particles adsorbed onto the droplets aggregate due to capillary attraction, indicating that the attractive capillary force is greater than the repulsive electrostatic force. Capillary interactions result from fluid surface deformations, which increase the surface area; reducing this excess area drives the particles closer together. The white dashed lines delineate the droplets and fluorescently labeled polystyrene particles appear green.

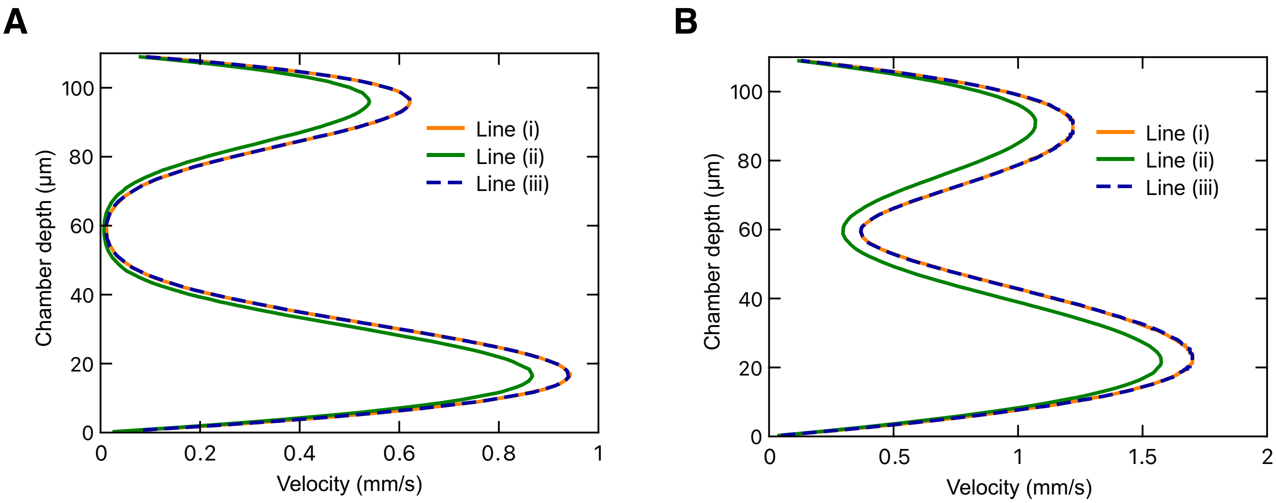

Figure S4: **Velocity profiles extracted from cut lines in Figure 5D with the no-slip (A) and slip (B) boundary conditions.** Velocities obtained with the slip boundary conditions are observed to be higher, although the trend of velocity variation remains consistent across both conditions. The results indicate that although velocities are slightly higher with the slip boundary condition, the distribution patterns and trends remain consistent with those observed with the no-slip boundary condition, where forces due to velocity gradients persist. To better reflect the physical reality, only simulation results obtained with the no-slip boundary condition are presented in the main text.

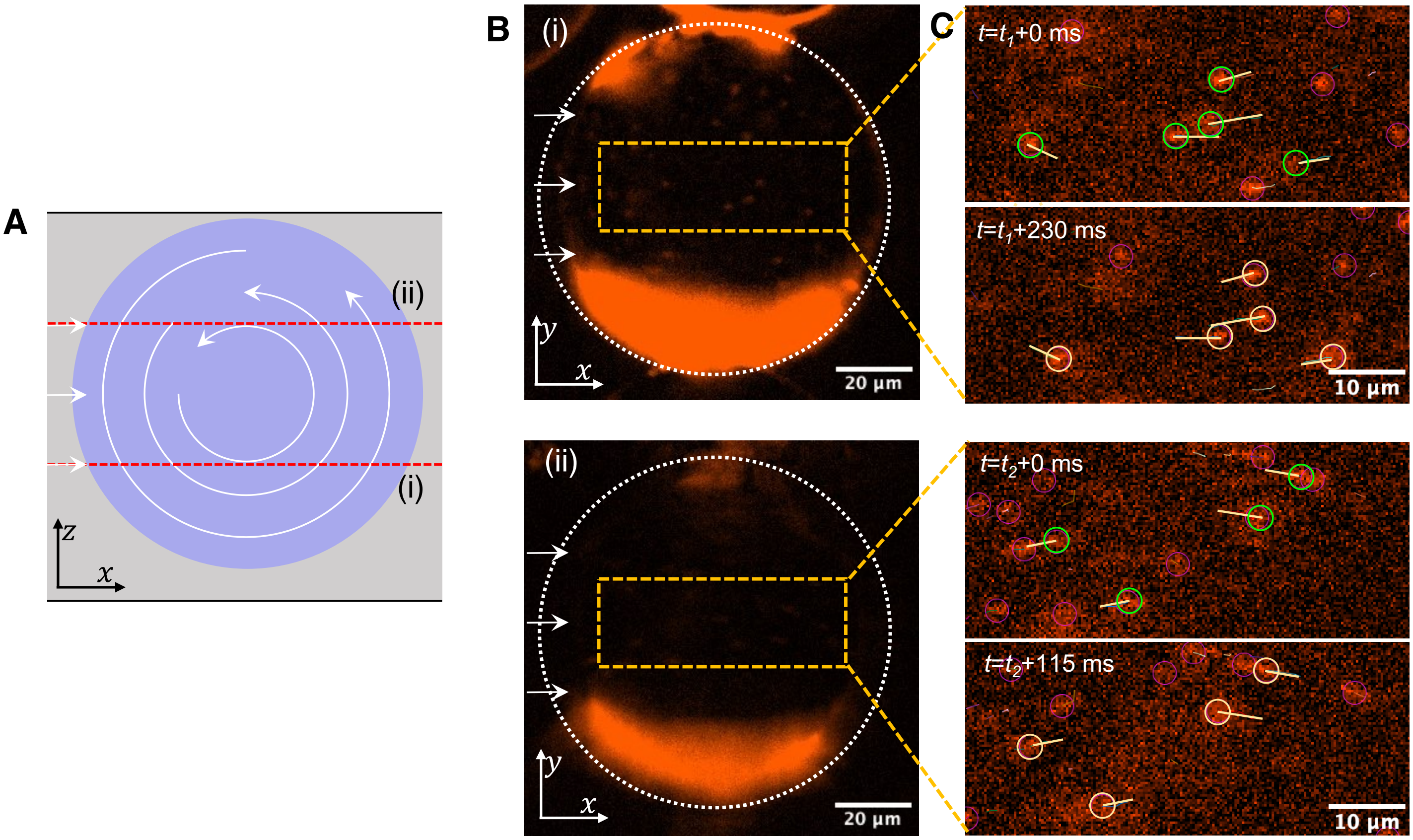

Figure S5: **Internal flow pattern of a droplet in response to the outer flow.** Internal flow within the droplets was visualized by introducing hydrophobic PMMA tracer particles. Despite the overlap in the fluorescence emission wavelengths between the PMMA and the polystyrene particles, the smaller size of PMMA particles enables us to distinguish their movement, thereby tracing the internal flow in the droplets. **A** Schematic diagram of the flow direction inside the droplet. **B** (i) The particles move from left to right with the focusing plane just below the center of the droplet. (ii) Conversely, the particles move from right to left with the focusing plane slightly above the center of the droplet. **C** Close-up views illustrate the positions of particles at different times, with yellow lines depicting their pathlines. Reconciling these observations with the numerical simulations, we conclude that the higher flow velocity in the lower part of the droplet induces a circulating flow, as schematically represented.

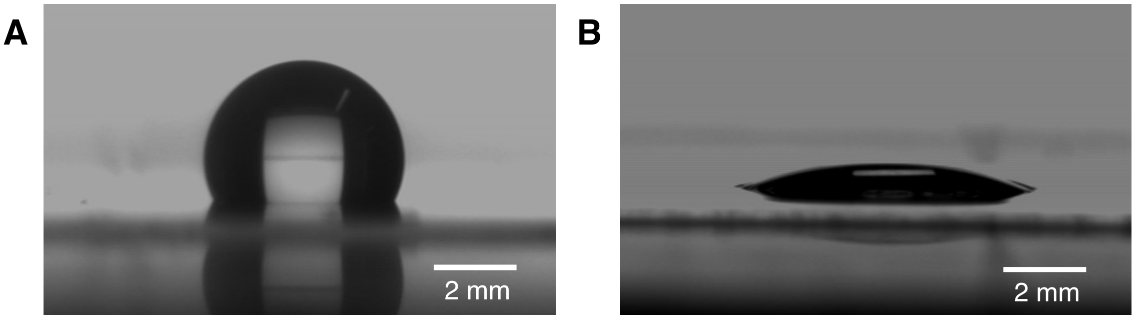

Figure S6: **Wettability of PDMS-coated cover glass.** **A** A 5 μL water droplet on an untreated PDMS-coated cover glass indicating a hydrophobic surface. The measured contact angle is $101.6 \pm 1.7°$, $N = 5$ independent droplets. **B** A 5 μL water droplet on a plasma-treated PDMS-coated cover glass stored in Milli-Q water and measured after 4 weeks showing a hydrophilic surface. The measured contact angle is $19.4 \pm 0.2°$, $N = 5$ independent droplets. A drop shape analyzer (DSA 100, KRÜSS) was used for measure the static contact angle.

3 Captions for Supplementary Videos

3.1 Supplementary Video 1: On-chip droplet generation and collection process

Droplets initially form at the flow-focusing junction and subsequently pass through a serpentine adsorption channel, where particles in the continuous phase penetrate and adsorb onto the droplet interfaces. Eventually, these particle-coated droplets enter the collection chamber. The particle concentration is 0.0625 wt%.

3.2 Supplementary Video 2: Particles flow past the droplets in collection chamber

Particles flow circumvent the droplet in the collection chamber rather than breaching and adsorbing at the droplet interface. Particles were visualized using fluorescence microscopy.

3.3 Supplementary Video 3: 3D reconstruction of collected droplets

3D reconstruction of a hexagonal packed droplet monolayer in the collection chamber was performed using multiphoton confocal microscopy images. The accompanying video illustrates the collected droplets rotating 360° around the Y-axis.

3.4 Supplementary Video 4: Droplet coalescence events

Particle-stabilized droplets within the collection chamber were monitored over a 12-hour period. During this time, three coalescence events were observed within the first seven hours, after which the droplets maintained stability until the video concluded.

3.5 Supplementary Video 5: Particle accumulation and circulation in response to the outer fluid flow

Particles adsorbed at the droplet interface accumulate at the poles, which are perpendicular to the external fluid flow direction. These particles also demonstrate circulation at these poles.

3.6 Supplementary Video 6: Internal flow within the droplet

Fluorescently labeled PMMA particles illustrate the direction of the flow within the droplet. The left video shows particles moving from left to right, with the focal plane just below the droplet's center, while the right video shows particles moving from right to left, with the focal plane positioned above the droplet's center. These observations collectively indicate a circulatory flow pattern inside the droplet.

References

[1] J. F. Wang, W. R. Hwang, *Journal of Composite Materials* **2008**, *42*, 9 909.

[2] F. Yang, Ph.D. thesis, King Abdullah University of Science and Technology, **2022**.

[3] COMSOL AB, Stockholm, Sweden.
